# When and how should we use imaging in individuals at risk of rheumatoid arthritis?

**DOI:** 10.3389/fmed.2022.1058510

**Published:** 2022-11-23

**Authors:** Kate Harnden, Andrea Di Matteo, Kulveer Mankia

**Affiliations:** Leeds Institute of Rheumatic and Musculoskeletal Medicine, University of Leeds, Leeds, United Kingdom

**Keywords:** ultrasound, rheumatoid arthritis, magnetic resonance imaging, computed tomography, at risk of rheumatoid arthritis, clinically suspect arthralgia, ACPA, palindromic rheumatism

## Abstract

In recent years rheumatologists have begun to shift focus from early rheumatoid arthritis (RA) to studying individuals at risk of developing the disease. It is now possible to use blood, clinical and imaging biomarkers to identify those at risk of progression before the onset of clinical synovitis. The use of imaging, in particular ultrasound (US) and magnetic resonance imaging (MRI), has become much more widespread in individuals at-risk of RA. Numerous studies have demonstrated that imaging can help us understand RA pathogenesis as well as identifying individuals at high risk of progression. In addition, imaging techniques are becoming more sophisticated with newer imaging modalities such as high-resolution peripheral quantitative computed tomography (HR-pQRCT), nuclear imaging and whole body-MRI (WB-MRI) starting to emerge. Imaging studies in at risk individuals are heterogeneous in nature due to the different at-risk populations, imaging modalities and protocols used. This review will explore the available imaging modalities and the rationale for their use in the main populations at risk of RA.

## Introduction

Rheumatoid arthritis (RA) is a chronic autoimmune disorder, which is characterized by poly-articular and systemic inflammation. It affects around 1% of the population and if poorly treated can lead to irreversible joint damage and disability (1). It is now widely accepted that early diagnosis and tight control of disease activity is associated with improved long-term outcomes (2). Subsequently the early phase of RA within 3 months of the development of synovitis, has been named the “window of opportunity.” Diagnosing and treating RA patients within this window can be difficult due to delays in patient presentation, referral delays and waiting times in secondary care (3). Furthermore, once RA has developed, drug free remission, which is effectively cure of the disease, remains infrequent (4, 5). This has led to a drive in identifying individuals at-risk of RA to offer the opportunity to treat prior to the onset of synovitis and potentially prevent or delay RA development.

A recent European League Against Rheumatism (EULAR) task force has used clinical features to define three main populations that should be considered when studying individuals at risk of RA (Table 1) (5, 6). These groups include asymptomatic predisposed individuals, individuals with positive serum auto-antibodies and early clinical arthritis. One specific group of frequently studied patients are those that have inflammatory MSK symptoms and they can be defined as having clinically suspect arthralgia (CSA) (7). Due to the American College of Rheumatology (ACR)/European League Against Rheumatism (EULAR) RA diagnostic criteria update in 2010, many patients who were previously diagnosed with undifferentiated arthritis (UA) would now be diagnosed with RA. As many of the imaging studies in UA recruited patients based on pre-2010 criteria, this review has not included imaging studies that have solely focused on UA patients.

**TABLE 1:** EULAR defined populations at risk of developing RA.

At-risk group	Subgroups
Asymptomatic individuals	• First degree relatives (FDRs) with RA • ACPA positive • Genetically predisposed indigenous populations
MSK symptoms	• Positive RA-related auto antibodies (Rheumatoid factor (RF), anti-citrullinated protein antibodies (ACPA) or Anti-carbamylated antibodies) • Clinically suspect arthralgia (CSA) Inflammatory clinical features such as difficult making a fist, a positive squeeze test or early morning stiffness (EMS) • Subclinical inflammation on imaging
Early clinical arthritis	• Undifferentiated arthritis (UA) • Palindromic Rheumatism (PR).

First group: Asymptomatic individuals with one of the following risk factors; a first-degree relative (FDR) with RA, positive serum anti-citrullinated protein antibodies (ACPA) or originating from a genetically predisposed indigenous population. Second group: Musculoskeletal (MSK) symptoms without clinical arthritis plus positive serum auto-antibodies (Rheumatoid factor (RF), ACPA or Anti-carbamylated antibodies) and/or have inflammatory clinical features such as difficult making a fist (8) or subclinical inflammation on imaging (CSA) (7). Third group: early clinical arthritis including undifferentiated arthritis (UA) and Palindromic Rheumatism (PR).

It is now accepted that many of these at risk individuals may be in a very early phase of what has been defined as the “RA disease continuum.” In those that do go on to develop RA, this phase can be retrospectively labeled as “pre-RA.” Many at risk individuals have biochemical and imaging abnormalities that can be used to predict progression to arthritis. These biomarkers can also provide a better understanding of the pathogenesis of the disease in the preclinical phase. A key point to note is that not all at risk individuals will progress to RA. It is therefore important to understand which biomarkers are the most useful in predicting progression.

The use of imaging in RA and other inflammatory arthritidies has increased dramatically in the past two decades. Previously it was mostly limited to the use of plain radiographs to detect irreversible joint damage in established RA. It has subsequently been shown that both Ultrasound (US) and MRI can be used to detect structural damage that is not visible on plain radiographs (9, 10) and subtle inflammation that is not detectable by clinical examination (11, 12). As well as US and MRI, HR-pQCT and molecular imaging techniques such as Position emission tomography (PET) have also shown promise in early RA (13, 14). This increased understanding and breadth of use of different imaging techniques is now being applied to individuals at risk of RA.

Interventional trials are now starting to focus on treating individuals at risk of RA in the preclinical stage. A recent randomized controlled trial (RCT) demonstrated that intervening with methotrexate and corticosteroids in CSA patients with subclinical inflammation on MRI can delay arthritis development and appears to be associated with a milder arthritis phenotype (15). Other RCTs using rituximab and abatacept have demonstrated that intervening in the preclinical stage could also delay and possibly prevent RA development (16, 17). The results of these studies should further our knowledge on the optimum timing and frequency to image individuals at risk of RA. They also demonstrate that halting the development of RA in the preclinical stage is now a realistic prospect. Imaging is likely to remain a central part of this process with its ability to help identify, stratify and manage individuals at risk of RA. Furthermore, patients anecdotally often relate better to images of their condition, allowing them to visualize the disease process, compared to numerical laboratory data. Patients report that undergoing scans is a positive experience and appreciate having the opportunity to view their images (18). In this review we aim to address how different imaging techniques should be used in individuals at risk of RA.

## Which imaging technique?

Multiple different imaging modalities have been used in individuals at risk of RA. There are advantages and disadvantages of each imaging method as discussed below (Table 2).

**TABLE 2:** A summary of the advantages and disadvantages of different imaging modalities and the evidence for the use in individuals at risk of RA.

Summary of imaging modalities					
	Advantages	Disadvantages	Use in at risk individuals	Predictive value	HR
Ultrasound	• Accessible • Low cost • No radiation • Easily tolerated • Superior to clinical examination at detecting synovitis (41)	• High operator dependency • Risk of false positives • Time consuming to assess multiple joints	Predicting the development of RA in symptomatic patients with autoantibodies and/or CSA (27, 28, 30)	**NPV 89%** for the development of RA in CSA (31)	**PD, HR 1.88–3.7** in ACPA+ with MSK symptoms (27, 28) **GS, HR 2.3** in ACPA+ with MSK symptoms (28)
MRI	• Multiplanar information on bone and soft tissue • Superior sensitivity to US in detecting synovitis and tenosynovitis (33, 35)	• Time-consuming • Expensive • Tolerability • Risk of false positives (specifically limited)	Predicting the development of RA in symptomatic patients with autoantibodies and CSA (41, 42, 55) MRI tenosynovitis is independently associated with IA progression in CSA and ACPA+ arthralgia (42, 55)	MRI inflammation **PPV 25–31%** and **NPV 93–96%** in CSA (41, 42) MRI Tenosynovitis **PPV 25%** and **NPV 95%** in CSA (41)	MRI synovitis **HR 1.08** in ACPA+ with MSK symptoms (55) MRI tenosynovitis **HR 4.02–8.39** in CSA and ACPA+ with MSK symptoms (42, 55)
PET	• Three dimensional imaging as well as functional imaging • Whole body imaging	• high cost • low availability • Ionizing radiation dose • specialist interpretation required	Potential use in predicting the development of RA in ACPA arthralgia patients (45)	N/A	N/A
CT	• Three dimensional imaging of the bone • Visualize early bone cortical changes including cortical microchannels	• Limited ability to assess soft tissues • Unable to detect inflammation • Ionizing radiation dose • HR-pQCT scans can be prone to motion artifacts	Predictive value for the development of RA with HR-pQCT by the detection of CoMiCs over metacarpal heads (51)	N/A	N/A
CR	• Low cost • Easily reproducible • Accessible	• Limited ability to assess soft tissues • Unable to detect inflammation	No evidence for use	N/A	N/A

ACPA+, anti-citrullinated protein antibodies; CoMiCs, cortical micro-channels; CT, computed tomography; CR, conventional radiography; CSA, clinically suspect arthralgia; GS, gray scale; HR-pQCT, high resolution peripheral quantitative computed tomography; MRI, magnetic resonance imaging; MSK, musculoskeletal; PET, positron emission tomography; PPV, positive predictive value; NPV, negative predictive value; PD, power Doppler; HR, hazard ratio.

### Ultrasound

The use of US in both research and clinical practice is now widespread in rheumatology. The benefits of US include accessibility, low cost, lack of radiation exposure and tolerability for patients. US is more sensitive than clinical examination for detecting synovitis (19) and its ability to differentiate synovitis from other causes of joint pain and swelling, such as tenosynovitis or bursitis, makes it an extremely useful tool in early disease, including individuals at risk of RA (20).

The potential disadvantages of US include the high operator dependency and therefore lower reproducibility. Another concern regarding individuals at risk of RA is that US may detect joint inflammation too late in the disease continuum, when clinical arthritis is imminent, therefore leaving limited opportunity for preventive intervention (21, 22). In line with this, when lower risk individuals have been studied, particularly those who have not developed joint symptoms, US abnormalities have not been found (23). Another concern is that not all patients with US inflammation will go on to develop RA (24). Joint effusions, synovial hypertrophy and even low level power Doppler (PD) signal can be commonly found in healthy populations (25). If a clinician finds US synovitis it may be tempting to start immunosuppressant medications which leads to the possibility of over-treating patients and potentially subjecting them to lifelong medications (26).

Despite these concerns, multiple studies have demonstrated the positive predictive value of US abnormalities in individuals at risk of RA. The initial US analysis from Leeds found that presence of US PD in the hands and wrists of CCP+ individuals with new MSK symptoms was associated with progression to IA (27). In a larger study from the same center, 136 ACPA positive patients with MSK symptoms were followed up over a median of 18.3 months. Fifty-seven (42%) patients developed an IA after a median of 8.6 months; 86% of patients that progressed to IA had one or more US abnormalities at baseline compared to 67% of patients that did not progress. Furthermore, US abnormalities were predictive of IA development at both patient and a joint level. Gray scale (GS), PD and erosions were all associated with progression, with PD conferring the highest risk. At joint level, the presence of PD at baseline was associated with a 10 fold risk of that joint developing clinical synovitis (28). In contrast, an US study in a Dutch seropositive arthralgia cohort found that GS synovitis was predictive of IA progression but PD was not (29). The contrasting results may be explained by cohort differences. This Dutch study included patients with lone RF positivity as well as ACPA positive patients, thus the overall cohort is lower risk. US was associated with progression to RA in a retrospective analysis of 80 patients with inflammatory arthralgia of < 6 weeks duration but negative rheumatoid autoantibodies. The Swiss Sonography Group in Arthritis and Rheumatism (SONAR) scoring system was used but PD was not included in the predictive analysis (30).

The negative predictive value of US has been specifically demonstrated in patients presenting with CSA with at least two painful joints of the hands, feet or shoulders. In a multicentre cohort study, US data was collected at baseline, 6 and 12 months. Fifty-nine percent of patients in this study who had US synovitis (defined as GS ≥ 2 and/or PD ≥ 1) at baseline developed IA. Importantly, if no joints showed US synovitis at baseline, the negative predictive value was 89%, suggesting that such individuals could be largely reassured of their risk of developing IA (31).

**FIGURE 1:**
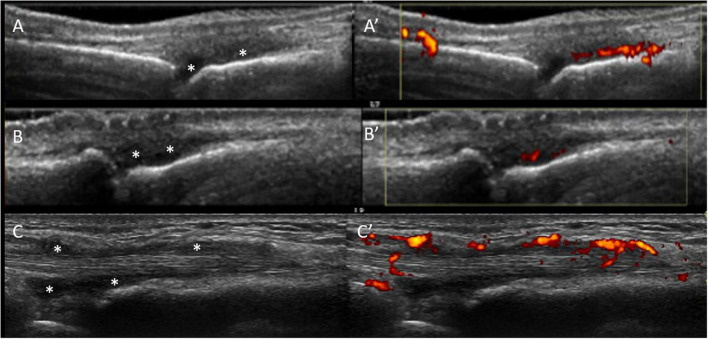
US sub-clinical synovitis and tenosynovitis in ACPA+ individuals with MSK symptoms. Gray scale **(A)** and power Doppler **(A’)** positive synovitis of the 3rd MCP joint in a patient at-risk of RA high titre positive anti-CCP antibodies and non-specific musculoskeletal symptoms. Similar US findings are shown in the 2nd PIP joint in a different at-risk individual **(B,B’)** with positive anti-CCP antibodies and rheumatoid factor, hands arthralgia. **(C,C’)** Illustrate tenosynovitis of the 2nd extensor tendon compartment in a third individual at-risk of RA with high titre anti-CCP antibodies and clinically suspect arthralgia. All images were obtained using a longitudinal approach. Asterisks indicate synovial hypertrophy.

The ability of US tenosynovitis to predict IA has been less well studied than with MRI and has shown mixed results. Molina Collada et al. found that in a cohort of CSA patients PD tenosynovitis at baseline was the only independent predictor of RA and IA development (32). In contrast van de Stadt et al. did not find that US tenosynovitis was significantly predictive of IA progression at joint or patient level (29). Again these contrasting results may be explained by cohort differences as the CSA patients in Molina Collada et al. paper had relatively high average RF and ACPA antibody titres. In a direct comparison of MRI and US, it was found that US was less sensitive than MRI in the early detection of both synovitis and tenosynovitis in patients with CSA (33). Figure 1 shows representative US findings of sub-clinical synovitis and tenosynovitis in ACPA+ individuals with MSK symptoms.

**FIGURE 2:**
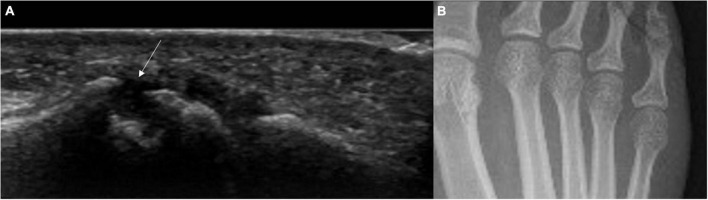
US bone erosion (not detected by x-rays) in the 5th MTP joint in an individual with high titre ACPA and non-specific MSK symptoms. **(A)** Longitudinal US scan of the lateral aspect of the 5th MTP joint. The white arrow indicates the presence of bone erosion. **(B)** Correspondent feet x-rays showing no bone erosions in the 5th metatarsophalangeal joint. This patient presented with non-specific musculoskeletal symptoms and high titre anti-CCP antibodies.

In a study that has looked at US detected bone erosions in “pre-RA” it was shown that bone erosions in the feet could be predictive for RA development. This was a large study that followed up 400 RA patients over a median of 41.4 months. Bone erosions in more than one joint and bone erosions in fifth MTP joint with US synovitis were the most predictive for the development of IA (34) (Figure 2).

In summary, US is a readily available imaging technique that provides valuable information in individuals at risk of RA. The presence of PD synovitis in symptomatic at risk individuals, is strongly associated with imminent future arthritis development and has been used to produce clinically relevant risk stratification models. Conversely, the value of US in at risk individuals without MSK symptoms appears to be limited.

### Magnetic resonance imaging

**FIGURE 3:**
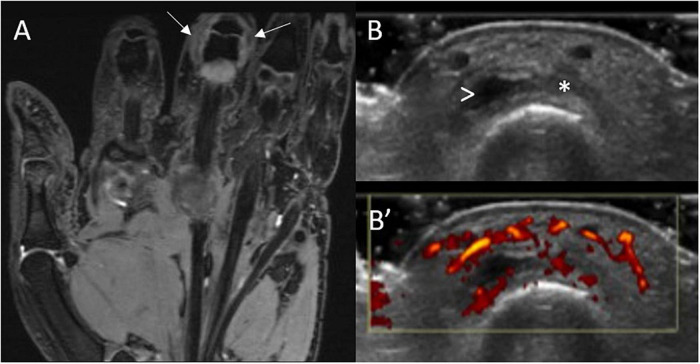
MRI and US sub-clinical synovitis in a patient with CSA and high titre ACPA. MRI **(A)** synovitis (white arrows) of the 3rd PIP joint in an individual at-risk of RA with corresponding gray-scale **(B)** and power Doppler **(B’)** US scans. This individual had clinically suspect arthralgia and positive anti-CCP antibodies (high titre) and rheumatoid factor. These images were obtained 6 weeks before progression to RA. Asterisk indicates synovial hypertrophy, while the arrowhead a small joint effusion.

One of the major benefits of MRI is its ability to provide highly sensitive multiplanar information on both the bone and soft tissue structures in and around the joints without using ionizing radiation. It has demonstrated superiority to US in detecting synovitis (Figure 3) and tenosynovitis in early RA and CSA (33, 35). This in addition to its unique ability to detect bone marrow edema, a potential precursor to erosions, makes its use in at risk individuals appealing (36). Despite this, MRI is not without its disadvantages; it is time-consuming, expensive and some patients struggle to tolerate it. Consequently US has generally gained more traction as the first line high resolution imaging assessment for synovitis in most rheumatology centers, with MRI often used as a second line investigation where required.

One specific concern with MRI in at risk individuals is that its high sensitivity for detecting inflammation may compromise specificity. MRI often detects inflammation in healthy, asymptomatic individuals without risk factors for RA (37, 38). One of the larger studies to investigate this performed contrast enhanced MRIs of the dominant metacarpophalangeal (MCP), wrist and metacarpophalangeal (MTP) joints of 193 symptom free persons. In this study, 72% of patients had at least one single inflammatory feature and 78% had one or more erosions. Inflammatory features and erosions were particularly prevalent in older age groups (39). In a small study of 28 patients with ACPA positive arthralgia, 93% of individuals had MRI synovitis with a Rheumatoid Arthritis Magnetic Resonance Imaging Score (RAMRIS) of 1. Forty-three percent of these patients went on to progress to IA and a RAMRIS score of 2 or above was associated with faster progression (40). Boer et al. created more specific parameters to define pathological MRI inflammation by comparison with a symptom free reference group. They demonstrated that by using a reference group MRI can be predictive in CSA and UA patients and the rates of false positives were reduced (41).

Larger studies of at risk individuals have demonstrated promising results in the predictive value of MRI. Van Steenbergen et al. looked at 150 patients with CSA of whom 46% had significant subclinical inflammation on MRI (synovitis, bone marrow edema or tenosynovitis) when scored against a healthy reference group. They followed up all patients up over a median of 75 weeks and 30% of patients developed IA. MRI inflammation was more positively associated with IA development than age, localization of initial symptoms and C-reactive protein level. Seventy-eight percent of the patients that had inflammation on MRI at baseline developed IA within a year compared with only 6% of patients without. Interestingly, tenosynovitis had the strongest independent association for progression to IA (HR = 7.56). Bone marrow edema and synovitis were also independently associated with progression but less strongly so (42).

Overall the current evidence suggests that MRI may have a role in assessing at risk individuals. It may have particular value in delineating inflammation in extra-capsular structures in a sub groups of patients, which requires further exploration. For practical reasons, faster and cheaper MRI protocols are likely to be needed before the use of MRI in clinical practice becomes more widespread.

### Positron emission tomography

Positron Emission Tomography (PET) is a nuclear imaging technique, which uses radioactive tracer drugs to detect metabolic cellular processes. It is used alongside another imaging technique, usually CT, to produce three dimensional functional imaging. PET-CT is able to detect synovitis and monitor treatment response in early RA (43). An important advantage is the ability to image the whole body in one acquisition unlike other more conventional imaging. Disadvantages include the high cost, limited availability (often restricted to large, specialist centers) and radiation dose. In addition, tracer uptake may not be specific to joint inflammation so specialist interpretation is required. Efforts are being made to improve safety; newer tracers have a much shorter half-life, which makes the radiation exposure similar to a standard CT scan (44). Moreover, the advent of PET MRI and the fact that PET scans are becoming increasingly sensitive is also likely to lower radiation exposure.

In their small pilot study, Gent et al. used (*R*)*-*^11^C-PK11195 PET to show that subclinical joint inflammation could be detected in 29 ACPA positive arthralgia patients. Hands and wrists were scanned at baseline and patients were then followed up over 24 months. Nine patients in total developed an IA. Four patients had a positive PET scan at baseline, all of whom went on to develop IA. Of the 5 patients who developed IA and had a negative scan at baseline, 3 of these patient developed inflammation in joints that were not scanned. These results of this preliminary study suggest that (*R*)*-*^11^C-PK11195 PET may be useful in predicting IA development (45).

Nuclear medicine is an evolving area in RA imaging. The evidence from PET and the potential to develop new radiotracers that can highlight areas of inflammation at whole body level warrants further exploration in individuals at risk of RA.

### Computed tomography

Unlike conventional radiography, CT provides three dimensional imaging of bone without projectional superimposition. However, unlike MRI, it has limited ability to assess soft tissues and is unable to detect inflammation. CT is also associated with ionizing radiation exposure, which, alongside the lack of information on soft tissue inflammation, has resulted in relatively little research into the use of CT in at risk individuals. There is evidence that changes in bone mineral density may begin in very early RA (46, 47). This has led to the question of whether some of these bone changes may occur in the “pre-RA” stage before the onset of clinical synovitis. HR-pQRCT is an imaging technique that was introduced over a decade ago and has shown promising use in individuals at risk of RA. One disadvantage is that it requires specialized technology that is not widely available. Kleyer et al. used a type HR-pQCT called microfocal CT (micro-CT) to investigate the association between ACPA and bone loss prior to the onset of inflammatory arthritis. They demonstrated that cortical bone thickness was significantly reduced in asymptomatic ACPA positive individuals compared to healthy controls (48). It is worth noting that cortical hand bone loss in early RA has been shown to predict radiographic hand joint damage (49). In contrast, in a separate study of 29 ACPA positive individuals trabecular bone was thinner when compared to controls but there was no significant difference with the cortical bone (50).

It is thought that erosions typically start in the “bare area” of a joint, which is not covered by articular cartilage. Simon et al. used HR-pQCT to investigate whether individuals at risk of RA have a higher frequency of cortical micro-channels (CoMiCs) at the bare joint areas. It was found that in 74 individuals, who were ACPA or anti-MCV positive, there were significantly more CoMiCs in the patients that progressed to RA and CoMiCs over metacarpal heads were associated with the development of RA (51). HR-pQCT scans have a higher spatial resolution than conventional CT scans with a similar radiation dose. A disadvantage is that they can be prone to motion artifacts. Currently HR-pQCT scans are not widely available for research or clinical purposes but their promising initial results in pre RA does warrant further investigation. Further research is needed into the use of CT in other at risk groups such as CSA, including ACPA negative individuals.

### Conventional radiography

Radiographs have been widely used in the initial work up of newly diagnosed RA patients and for the monitoring of disease progression. While they do not provide any information on soft tissues or synovial inflammation, they can detect structural joint damage. The benefits of radiographs include their low cost, accessibility and reproducibility for serial assessment. However, it has been shown that radiographs have limited ability in detecting bone erosions in early RA (52, 53). This clearly limits their use in predicting disease progression in at risk individuals. In a study of 418 ACPA positive at risk individuals only 4.1% had bone erosions on hand and feet radiographs and these did not predict progression to IA (54). This study suggests there is no value in routinely performing radiographs in individuals at risk of RA.

## Should we image extra-capsular structures?

As discussed above, intra-articular joint inflammation identified on US, MRI and PET-CT in at-risk individuals is associated with progression to IA. However, it is not only the joints but also the structures outside the joint capsule that have shown interesting findings in individuals at risk of developing RA. MRI tenosynovitis is a particularly important finding as evidenced by Van Steenbergen et al. who found it to be the strongest independent predictive factor on MRI for the development of IA in patients with CSA (42). Further studies in ACPA positive individuals have also demonstrated that tenosynovitis is the strongest MRI predictor of progression to IA (55, 56). A recent study in CSA patients found that MCP-extensor peritendinitis, although infrequent, was strongly associated with IA development with a positive predictive value of 65% (57). Similarly, MRI interosseous tendon inflammation was identified in 19% of ACPA positive patients, 49% of early arthritis patients but no healthy controls (58). A histological study confirmed the absence of a tenosynovial sheath around the interosseous tendons, suggesting the MRI findings reveal a peri-tendinous inflammation rather than a genuine tenosynovitis. While US tenosynovitis has more mixed findings in predicting progression, it is important to note that when present it is highly likely to be pathological; it is infrequently seen in healthy individuals (59).

Other extracapsular structures are also of relevance in at risk individuals. Non synovial extra-capsular inflammation, in the absence of synovitis, represents a distinct phenotype in PR patients during flare (60). A very recent study found that inter-metatarsal bursitis may precede the development of RA. In this study, contrast enhanced MRI scans were performed in the forefoot, MTP and wrist joints of 577 CSA patients. The RAMRIS scoring system was used and intermetatarsal bursitis was only counted as being present if it would be uncommon in the same location in a healthy population. They found that 23% of CSA patients had intermetatarsal bursitis but this increased to 47% if only including the ACPA positive patients. In the ACPA positive patients, intermetatarsal bursitis was able to predict progression to IA (61).

Overall, these studies have demonstrated the relevance of MRI inflammation in extracapsular structures in at risk individuals. Although of pathobiological relevance, further research is required to determine if imaging these extracapsular structures adds additional value in predicting progression to RA in at risk individuals.

## Can we image fewer joints?

Practically it is important to address how many and which joints should be scanned in individuals at risk of developing RA. To date, the majority of imaging studies in this cohort have used comprehensive imaging protocols that include a large number of joints. While this may be feasible in a research setting it is not usually practical in a clinical setting where time is limited.

One study that looked at a reduced subset of 30 unilateral RA specific MRI features in the wrist, MCP and MTP joints, as opposed to the 61 features in the RAMRIS scoring system, found that by using this smaller subset of measurements it was still possible to predict the development of arthritis in 225 CSA patients (62). In the Leeds cohort of ACPA positive patients with MSK symptoms, an US protocol of 32 joints was used to successfully predict progression to RA (28). Gray scale, PD and erosions were all shown to separately predict progression. In the first Leeds prediction model, presence of PD signal in 22 joints (the wrists, MCPJs and PIPJs only) was predictive of progression on multivariable analysis (27). This study demonstrated that an attenuated joint set of the hands and wrists only can have predictive value. van de Stadt et al. took a different approach in their study and only scanned tender joints and small joints directly adjacent and contralateral to the tender joints. It was found that in the 192 individuals with arthralgia and positive autoantibodies (RF and/or anti-CCP), only GS on US was predictive (29). As previously discussed, the different results in these studies may also be partly explained by differences in the at risk cohorts.

To date, US studies in individuals at risk of RA have all used bilateral joint protocols. In established RA it has been shown that unilateral reduced scoring protocols of 7–9 joints were still able to capture 78–85% of the information from a full 36 joint protocol. This was, however, significantly less than the bilateral 7–9 joint protocols which captured 89–93% of the information (63). In contrast, MRI protocols in individuals at risk of RA have used the most symptomatic or dominant hand and wrist joints, as recommended in the RAMRIS scoring system (64). One concern with this approach is that synovitis does not always present symmetrically. In a study that looked at early RA patients it was shown that 21% of patients had unilateral synovitis in non-dominant joints (65). Furthermore, by just scanning the dominant hand and wrist joints it allows the potential of overuse tenosynovitis to influence findings (66). Despite these concerns, multiple MRI studies have demonstrated predictive value of imaging only the dominant hands in individuals at risk of RA (40–42).

RA is often considered a disease of the small joints, with large joints affected less frequently and later into the disease course (67, 68). This leads to the question of whether large joints should be included in imaging protocols of at risk individuals. Rogier et al. found that in 170 CSA patients scanning the shoulders did not predict the development of IA despite 50 patients showing abnormalities on the US scan. However, only 5% of shoulders scanned in this study were symptomatic (69). In an MRI study of 55 individuals with ACPA and/or RF antibodies it was found that MRI and synovial biopsy of the knee did not detect clear-cut inflammation in the 15 patients who went on to progress to RA (70). In contrast, when ACPA+ at risk individuals had symptomatic knees and shoulders, performing US in these areas added predictive power (28). As such, a pragmatic approach may be to scan all standard protocol small joints but only include the symptomatic large joints.

Overall, an attenuated US joint set and a reduced scoring system for MRI can have predictive value in individuals at risk of developing RA. Unilateral US protocols have not been investigated in at risk populations. However, given a significant amount of information is lost with unilateral protocols in established RA it seems likely that bilateral joint assessments should be retained. In MRI the use of the most symptomatic or dominant hand and wrist joints is effective in predicting progression in individuals at risk of RA. Scanning symptomatic large joints on US adds predictive power. Imaging techniques such as PET (45) and whole body MRI (WB-MRI) that are able to image the whole body in one acquisition may also aid in the dilemma of which joints to scan. As far as we are aware, WB-MRI is yet to be evaluated in at risk individuals but its ability to visualize total patient-level inflammatory burden may warrant further investigation in at risk individuals.

## Hands, feet or both?

One limitation of US imaging of the feet in at risk individuals is that US abnormalities such as gray-scale synovial inflammation are fairly prevalent in the healthy population, particularly at MTPJ1 (25). The SONAR score includes the same joints as the DAS28 but excludes the thumbs, shoulders and also excludes the feet. Zufferey et al. found US to be predictive for IA development when using the SONAR score in 80 CSA patients (30). In contrast, Brulhart et al. did not find that a baseline SONAR US score predicted progression to RA. The cohort in their study, however, largely consisted of FDRs with mostly negative autoantibodies, and hence had a lower overall risk of progression, although interestingly 70% were symptomatic (23). Rakieh et al. also demonstrated that US of the hands and wrists alone can be predictive of progression in ACPA positive patients with MSK symptoms (27). Taken together, these studies demonstrate that in higher risk populations with MSK symptoms, US protocols that do not include the feet can still provide predictive information. However, it is not clear to what extent omitting the feet has an effect on predictive accuracy. For example, a recent study demonstrated useful additional information to be gained by including the feet in US protocols of individuals as risk of RA. A baseline US scan was performed in over 400 ACPA positive individuals to evaluate bone erosions in MCP2, MCP5 and MTP5 joints. The combination of bone erosions and synovial inflammation in MTP5 was the most successful in predicting RA development compared with the combination of synovial inflammation and erosions in either MCP2 or MCP5 (34).

One MRI study has addressed the importance of including the feet alongside the hands when imaging at risk individuals. Boer et al. performed contrast enhanced MRI of the hand (MCP2-5 and wrist) and foot (MTP1-5) in 357 CSA patients. Scans were scored for synovitis, osteitis and tenosynovitis. After 1 year follow up 18% of patients developed an IA. The investigators found that although tenosynovitis of the feet could independently predict IA it did not increase the overall predictive accuracy of MRI over the hands and wrists alone. Without including the feet, the overall predictive sensitivity remained at 77%, however, the specificity actually decreased from 66 to 62% (71).

In summary, there is evidence to suggest specific benefit from including the feet in US protocols for at risk individuals. Therefore, reducing the length of protocols by using a limited set of hand and foot joints may be the best approach for improving feasibility while retaining predictive accuracy. In MRI, unvalidated data suggests imaging the most dominant or symptomatic hand and wrist joints alone without the feet is sufficient to predict progression to RA. A summary of suggested structures to image for MRI and ultrasound is included in Table 3.

**TABLE 3:** A summary of which structures to image with MRI and US in individuals at risk of RA.

	Structures to image that add predictive value	Structures to image for diagnostic/Pathological value
US	• Bilateral attenuated subset of small joints (27, 28) • Bone erosions in the feet (34) • Symptomatic large joints (28)	• Extracapsular inflammation in PR (60) • Tendons (59)
MRI	• Most symptomatic or dominant hand and wrist joint (40–42) • Tendons of the hands (42, 55, 56) • Intermetatarsal bursae (61)	• Interosseous tendons (58) • Tendons of the feet (71)

The structures that add predictive value in ultrasound (US) include the small joints, which can be an attenuated subset, bone erosions in the feet and symptomatic large joints. US of tendons adds diagnostic and pathological value as does extracapsular inflammation in palindromic rheumatism (PR) patients. The structures that add predictive value in magnetic resonance imaging (MRI) include the most symptomatic or dominant hand and wrist joint, tendons of the hands and intermetatarsal bursae. MRI of interosseous tendons and tendons of the feet adds diagnostic/pathological value.

## Which at risk populations should be imaged?

Populations who may be considered “at risk” of developing RA have now been defined by EULAR (6). These groups encompass a range of risk and symptom profiles, with some having a much higher risk of progression than others. Imaging can be time consuming and expensive, and also necessitates face to face clinical visits, so it is important to understand in which at risk populations it adds value.

Some people are at risk of developing RA despite having no MSK symptoms, e.g., asymptomatic genetically predisposed individuals. In terms of US studies, only 14.9–33% (27–29, 31) of patients with MSK symptoms have US PD on their baseline scan. This leads to the question of whether it is useful to image at risk individuals without MSK symptoms, as intuitively they may be even less likely to have subclinical inflammation on imaging. Only one study has looked at imaging individuals at risk of RA who do not have symptoms. Brulhart et al. performed US assessments using the SONAR score in 273 FDRs of RA patients of whom 8% were ACPA positive; 14% asymptomatic, 55% MSK had symptoms and 21% had UA. A positive US was defined as at least one joint with GS ≥ 2, or PD ≥ 1. US positivity was only found in the patients that had UA and not in the individuals that were asymptomatic regardless of their antibody status (23).

Seronegative patients with inflammatory symptoms (e.g., CSA) are a lower risk group for progression to RA than ACPA+ individuals with MSK symptoms. In CSA patients, MRI studies have shown that certain symptoms in particular are associated with subclinical inflammation. In their study of 575 CSA patients Krijbolder et al. found that the longer the duration of morning stiffness the more frequently subclinical inflammation was found on MRI (72). Only 14% of these patients were ACPA positive and 20% were RF positive. Further studies of MRI scans on CSA patients with similar antibody prevalence have shown that difficulty making a fist is associated with flexor tenosynovitis and a positive squeeze test is associated with subclinical synovitis (73, 74). Van der Ven et al.’s study included 143 CSA patients of whom only 13% were positive for ACPA and 26% for RF. In these patients the presence of US synovitis was still associated with IA development despite the lower antibody prevalence (30, 75). When patients do go on to develop RA around 25% of these patients are seronegative. These studies indicate the importance of imaging in patients who are seronegative, but have inflammatory MSK symptoms such as CSA.

Overall, it seems prudent to perform imaging preferentially in at-risk individuals who have MSK symptoms, even if they are autoantibody negative. Performing US scans in individuals without MSK symptoms may not add value, although US data is limited to a single study and it is unknown if this is the case with all imaging techniques. Clarity is also required on whether individuals with high autoantibody titres and non-MSK symptoms (e.g., fatigue) have subclinical inflammation on imaging in the absence of MSK symptoms such as joint pain and stiffness. A recent prospective observational study found that 21% of 92 asymptomatic ACPA positive individuals developed RA after an average of 10.7 months (76). This relatively high proportion of progression suggests that there may be value in imaging certain high risk asymptomatic individuals.

### Palindromic rheumatism

Palindromic rheumatism (PR) is a syndrome characterized by interment flares of joint pain and swelling. Patients are asymptomatic between flares and many have positive autoantibodies with 42–67% ACPA positive and 42–82% RF positive (60, 77–80). PR was included in the EULAR defined at-risk populations as a significant number of patients with PR go on to progress to RA (79, 81).

Given flares of PR are transient and unpredictable, imaging can be practically challenging. A study that scanned 54 PR patients between flares found that only 7.4% had US subclinical synovitis in the asymptomatic phase (77). It is worth noting that the majority of PR patients in this study were not DMARD naïve which may have affected the imaging findings. However, a further study in DMARD naïve PR patients also did not find US inflammation between flares (60). In contrast when US scans were performed in symptomatic flares of 84 PR patients, 36% had synovitis on imaging (82). Seropositive patients were more likely to have US detected synovitis in flare. In this same cohort it was shown that US along with ACPA antibody status were able to successfully predict RA development within 3 years, although it was the ACPA status that was the most predictive (83).

PR patients have a distinctive imaging phenotype during flare (60). In a study of 31 treatment naïve PR patients it was found that 61% of patients had extra-capsular inflammation during flares. In 39% of the patients there was extracapsular inflammation on imaging without associated synovitis. Only 23% had US detected synovitis during flare. This distinct imaging phenotype of isolated extracapsular inflammation may be particularly useful in differentiating PR from RA on clinical assessment. Overall, the current evidence suggests that PR patients should be imaged during a flare and not when they are in the asymptomatic phase.

## Conclusion

The ability to study at risk individuals before they develop RA has opened up the possibility of a new and earlier “window of opportunity” for treatment. The implication of this is that there is now very real potential to treat prior to arthritis development with the prospect of halting disease progression. It is clear that imaging has a role in this group of individuals in differential diagnosis and risk stratification.

So far, MRI and US have been the most investigated imaging techniques in individuals at risk of RA and studies have shown useful outcomes. MRI may be optimum for certain inflammatory parameters such as tenosynovitis, however, US represents the safest, cheapest and most practical imaging tool. Newer imaging techniques such as HR-pQRCT and PET have shown promising initial results and warrant further investigation.

Further work is needed to establish the optimum imaging protocols that give the most accurate and efficient results. Current studies have demonstrated that it is possible to design MRI and US protocols with reduced joint numbers. In US in particular there does appear to be additional benefit in imaging the feet and symptomatic large joints. Extra-articular structures can also provide additional information. In MRI, imaging the tendons and intermetatarsal bursa in particular can inform risk stratification. US extracapsular inflammation in PR patients may be beneficial in differentiating PR from early RA.

**FIGURE 4:**
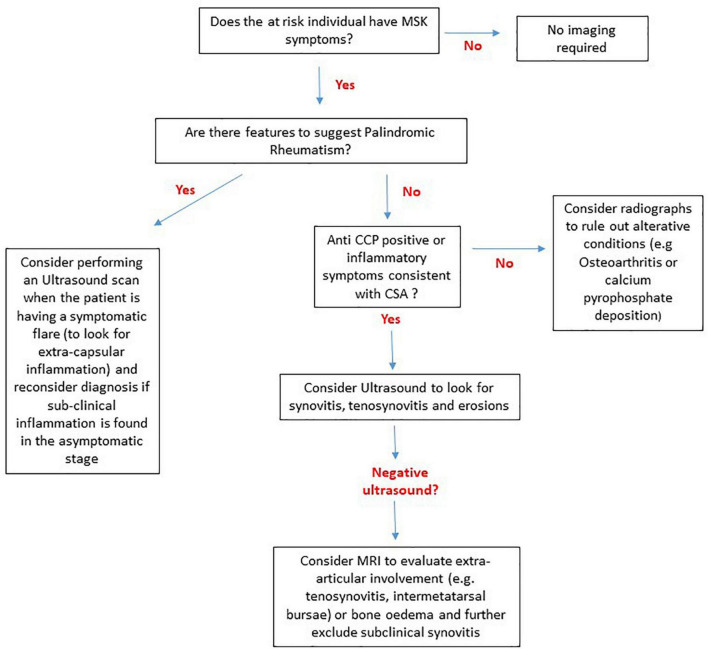
A suggested algorithm to guide the use of MRI, US and radiography in individuals at risk of RA without clinical synovitis based on current evidence. In patients that do not have any symptoms there is no evidence to suggest that any imaging techniques are of diagnostic or predictive value. For Palindromic rheumatism extracapsular inflammation can be captured on imaging during flare episodes and if subclinical inflammation is seen in the asymptomatic phase the diagnosis of palindromic rheumatism should be re-considered. For anti-citrullinated protein antibody (ACPA) negative individuals with inflammatory symptoms i.e., clinically suspect arthralgia (CSA) and ACPA+ individuals an ultrasound should be performed. If the ultrasound is negative MRI may be able to detect subtle sub-clinical inflammation, particularly in extracapsular structures. For at risk individuals such as first-degree relatives with non-inflammatory musculoskeletal (MSK) symptoms radiographs should be performed to look for alterative diagnoses.

A careful clinical history in individuals at risk of RA is important to guide the use and timing of imaging. Symptomatic at risk individuals should be scanned preferentially regardless of their antibody status. In PR patients, it is more valuable to scan patients during a flare than in the asymptomatic phase. It should be noted that in certain lower risk groups such as CSA patients with low ACPA antibody prevalence, US inflammation appears to be less frequent and MRI may add more value. Further research is needed to establish whether there is value in imaging asymptomatic individuals with high antibody titres and other risk factors. A suggested algorithm to guide the use of imaging in at risk individuals is suggested in Figure 4.

With management strategies in early RA moving to a more personalized and preventative approach, risk stratification models which include serological, cellular and imaging biomarkers are being increasingly formulated. It is therefore essential that we continue to optimize the use different imaging techniques within this important cohort.

## Author contributions

KH, AD, and KM contributed to the literature review and drafting of the manuscript. All authors contributed to the article and approved the submitted version.
